# Transgenic Chicks Expressing Interferon-Inducible Transmembrane Protein 1 (IFITM1) Restrict Highly Pathogenic H5N1 Influenza Viruses

**DOI:** 10.3390/ijms22168456

**Published:** 2021-08-06

**Authors:** Mohammed A. Rohaim, Mohammad Q. Al-Natour, Mohammed A. Abdelsabour, Rania F. El Naggar, Yahia M. Madbouly, Kawkab A. Ahmed, Muhammad Munir

**Affiliations:** 1 Division of Biomedical and Life Sciences, Faculty of Health and Medicine, Lancaster University, Lancaster LA1 4YG, UK; m.a.rohaim@lancaster.ac.uk (M.A.R.); m.al-natour@lancaster.ac.uk (M.Q.A.-M.); 2 Department of Virology, Faculty of Veterinary Medicine, Cairo University, Giza 12211, Egypt; 3 Department of Veterinary Pathology & Public Health, Faculty of Veterinary Medicine, Jordan University of Science and Technology (JUST), P.O. Box 3030, Irbid 22110, Jordan; 4 Department of Poultry Viral Vaccines, Veterinary Serum and Vaccine Research Institute (VSVRI), Agriculture Research Centre (ARC), Cairo 11435, Egypt; dr.m.adel.vsvri@gmail.com (M.A.A.); yahia.m.madbouly@gmail.com (Y.M.M.); 5 Department of Virology, Faculty of Veterinary Medicine, University of Sadat City, Sadat 32897, Egypt; rania.elnagar@vet.usc.edu.eg; 6 Department of Pathology, Faculty of Veterinary Medicine, Cairo University, Giza 12211, Egypt; kawkababdelaziz@yahoo.com

**Keywords:** transgenic chickens, chIFITM1, HPAIV H5N1, zoonotic infections

## Abstract

Mammalian cells utilize a wide spectrum of pathways to antagonize the viral replication. These pathways are typically regulated by antiviral proteins and can be constitutively expressed but also exacerbated by interferon induction. A myriad of interferon-stimulated genes (ISGs) have been identified in mounting broad-spectrum antiviral responses. Members of the interferon-induced transmembrane (IFITM) family of proteins are unique among these ISGs due to their ability to prevent virus entry through the lipid bilayer into the cell. In the current study, we generated transgenic chickens that constitutively and stably expressed chicken IFITM1 (chIFITM1) using the avian sarcoma-leukosis virus (RCAS)-based gene transfer system. The challenged transgenic chicks with clinical dose 10^4^ egg infective dose 50 (EID_50_) of highly pathogenic avian influenza virus (HPAIV) subtype H5N1 (clade 2.2.1.2) showed 100% protection and significant infection tolerance. Although challenged transgenic chicks displayed 60% protection against challenge with the sub-lethal dose (EID_50_ 10^5^), the transgenic chicks showed delayed clinical symptoms, reduced virus shedding, and reduced histopathologic alterations compared to non-transgenic challenged control chickens. These finding indicate that the sterile defense against H5N1 HPAIV offered by the stable expression of chIFITM1 is inadequate; however, the clinical outcome can be substantially ameliorated. In conclusion, chIFITM proteins can inhibit influenza virus replication that can infect various host species and could be a crucial barrier against zoonotic infections.

## 1. Introduction

The interferon-inducible transmembrane proteins (IFITMs) are a family of small transmembrane proteins induced by interferon (IFNs) and mount a profound antiviral state against multiple viruses [1]. The IFITM proteins restrict viral infections by blocking the viral entry and restrict the fusion of the viral and host membranes, thereby interfering with viral entry and replication [2,3]. It has been shown that IFTIM1, 2, and 3 are immune-related genes, critically involved in immune defense against a variety of viruses, including influenza virus, dengue virus, filoviruses, coronavirus, hepatitis C virus, lyssaviruses, and West Nile virus [4,5,6,7,8].

IFITM genes belong to a wider family known as dispanins with a common transmembrane domain configuration [5]. The IFITMs are genetically well characterized in vertebrates, and homologs are known to be present in bacteria [9] while IFITMs in birds have been given limited attention. IFITM proteins contain N- and C-termini, two transmembrane domains, and a conserved cytoplasmic domain [10]. IFITM1 has a shorter N-terminal region and is found on the periphery of cells and early endosomes [5]. Chicken IFITM (chIFITM) locus is clustered on chromosome 5 and contains five genes, namely, *chIFITM1*, *2*,* 3*,* 5*, and *10* [7,11]. The clustering profiles of gene expression reported the anti-viral response for IFITM1 and IFITM2 while IFITM3 action might be before fusion of viral membrane leading to viral entry blockage [7]. Previous studies have shown that host responses to avian influenza infection have varied significantly from chickens and ducks [7]. The IFITM1, 2, and 3 are strongly upregulated in response to highly pathogenic avian influenza virus (HPAIV) infection in ducks, whereas little response was seen in chickens [7]. In vitro overexpression of *chIFITM1* has been shown to increase the resistance of avian cells to AIV infection [7].

Highly pathogenic avian influenza viruses (HPAIVs) are causing devastating economic and welfare impacts on poultry and have significant human health implications around the globe with concerns on the emergence of new strains that lead to pandemics [12]. Understanding the host factors related to the virus’s pathobiology in their natural hosts may help to develop effective intervention strategies and define the genetic markers for disease resistance. Genetic analysis has suggested that host restriction factors play a major role for influenza virus replication [13]. However, only recently have the molecular functions and mechanisms been unraveled. Interactions between viral proteins and host factors are generally thought to play a major part in viral fitness and pathogenicity, and adaptive virus mutations lead to optimum interaction with host factors [14].

To map host restriction factors that determine the zoonotic potential and pathobiology of influenza viruses, we generated transgenic chickens that express chIFITM1 using the avian sarcoma-leukosis virus (RCAS)-based gene transfer system. The present study shows that chIFITM1 can inhibit H5N1 HPAIV at the clinical challenge dose while improving the clinical outcome of a sub-lethal challenge dose, which provides proof of an inhibition of the spread of zoonotic viruses to humans by virus resistant transgenic chickens.

## 2. Results

### 2.1. Efficient Expression of chIFITM1 Using RCAS Vector System

**Figure 1:**
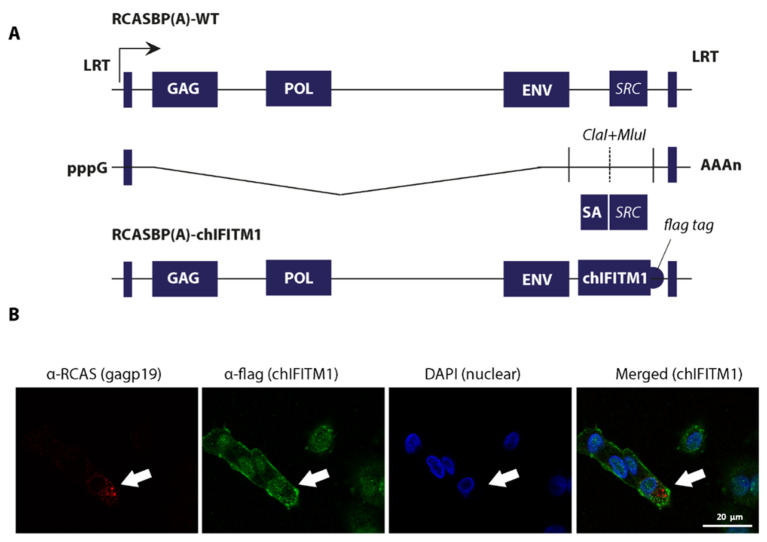
Generation and rescue of recombinant retroviruses expressing chIFITM1. (**A**) A schema for the generation of recombinant RCASBP virus in which src gene was replaced with chIFITM1. (**B**) Retroviruses were rescued in DF-1 cells and stained for retroviral structural gag protein and flag-tagged fused to the chIFITM1.

In order to determine the in vivo antiviral ability of chIFITM1 protein against avian influenza virus subtype H5N1, we generated transgenic chickens stably expressing chIFITM1 protein. To achieve this transgenesis, we exploited avian retroviruses (RCAS; Replication Competent ALV LTR with a Splice acceptor) vector-based expression system to generate mosaic transgenic chicken [15,16]. The full-length open reading frame of chIFITM1 was cloned between two unique restriction sites to efficiently express a caped and poly-adenylated transcript (Figure 1A). Correspondingly, RCASBP(A)-WT was used as negative control in the transgenesis process. Both RCASBP(A)-chIFITM1 and RCASBP(A)-WT recombinant viruses were rescued using chicken embryo fibroblasts (DF-1) to generate mosaic-transgenic chicken embryos for constitutive expression of chIFITM1. The virus replication was assessed by immunofluorescence staining for the viral structural protein (gag) and flag-tagged chIFITM1 by confocal microscopy indicating stable expression of the protein (Figure 1B). Infectious DF1 cells expressing RCAS-mediated chIFITM1 were further expanded to obtain the required stock density for transgenic embryo generation.

### 2.2. Generation of Transgenic Chicks Expressing chIFITM1

**Figure 2:**
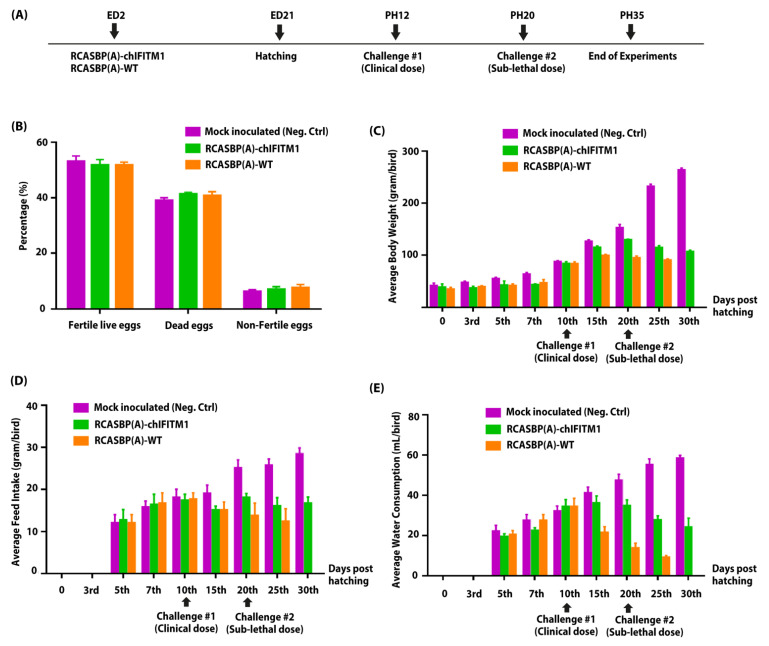
Generation of transgenic chickens and impact of chIFITM1 on hatchability and physiological parameters of hatched chicks. (**A**) Schema representing the time of transgenesis and challenge experiments. Comparison of hatchability percentage (**B**), body weight (**C**), feed intake (**D**), and water consumption (**E**) of chIFITM1-expressing transgenic chicken and control chicks (RCASBP(A)-WT and negative control) post-hatching (PH), pre-challenge and post-challenge. Statistical analyses between different inoculated groups were provided within Supplementary Data files 1, 2, 3, and 4. Values of *p* value < 0.05 were considered statistically significant.

For generation of mosaic transgenic chickens, 2-day-old embryonated SPF eggs were inoculated with recombinant RCAS viruses (RCASBP(A)-chIFITM1 or RCASBP(A)-WT) infected DF1 cells (Figure 2A). The hatched chicks were kept in isolators until challenge with clinical dose of HPAIV H5N1 at 12 days of age, and sub-lethal dose on day 20 of chick’s age (8 days post first infection) (Figure 2A). In two independently performed experiments, we confirmed that the chIFITM1 expression did not have any detrimental effect on the chick’s embryonic development and hatchability of RCAS-chIFITM1 transgenic eggs compared to mock groups (Figure 2B; Supplementary data file 1). In addition, it was noted that all transgenic RCASBP(A)-chIFITM1 or RCASBP(A)-WT chicks had a non-significant body weight reduction (Figure 2C; Supplementary data file 2) and progressively regained their body weight until equal to weights of mock inoculated group (negative control, inoculated with PBS) on the 10th day post-hatch (Figure 2C). All chicks, regardless of nature of transgenesis either with RCASBP(A)-chIFITM1 or RCASBP(A)-WT, ate (Figure 2D; Supplementary data file 3) and drank (Figure 2E; Supplementary data file 4) equally comparable to the negative-control group indicating general growth parameters.

### 2.3. Challenge Experiments and In Vivo Efficacy of chIFITM1 against Challenge with Clinical and Sub-Lethal Doses of HPAIV

There is a direct correlation between the infectious virus dose and the severity of the clinical infections. Therefore, the nature of HPAIV H5N1 virus and host genetics determine the clinical outcome of infection [17,18]. It was critical to determine the inoculum titer of HPAIV H5N1 that was able to induce clinical disease in chickens. Based on our previous study, we used the pre-optimized doses 10^4^ EID_50_ (hereafter called clinical) and 10^5^ EID_50_ (hereafter called sub-lethal) of HPAIV H5N1 strain A/chicken/Egypt_128s_2012 (clade 2.2.1.2) (accession number: JQ858485.1) [13,19,20] as a challenge virus to demonstrate the antiviral potential of chIFITM1 in transgenic chicks.

**Figure 3:**
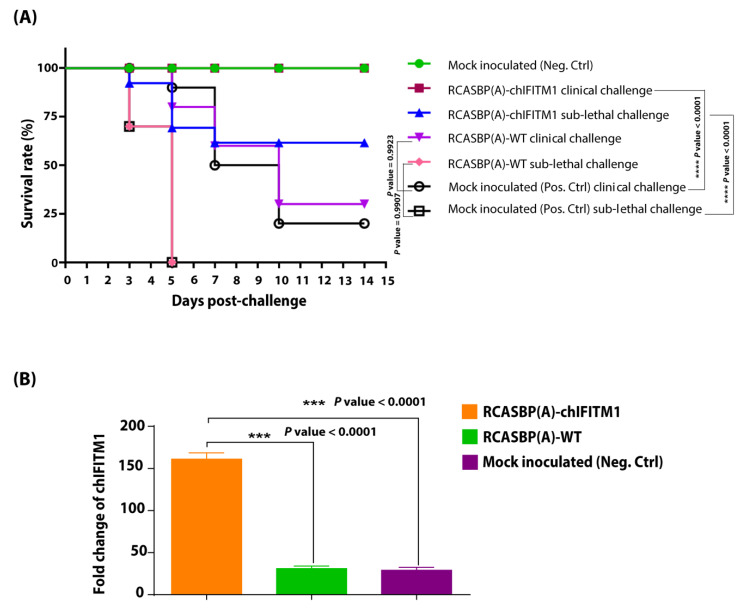
Survival rates and chIFITM1 expression quantification. (**A**) Percentage survival rates of RCASBP(A)-chIFITM1 and RCASBP(A)-WT challenged chicks with clinical and sub-lethal doses of H5N1 HPAIV compared to mock inoculated chicks (negative and positive control groups). (**B**) Expression of chIFITM1 in HPAI H5N1 challenged transgenic chickens with RCASBP(A)-chIFITM1 compared to transgenic chickens (RCASBP(A)-WT) and non-transgenic chicken (mock inoculated neg. ctrl), asterisks indicate significant difference.

Interestingly, the transgenic chicks expressing chIFITM1 when challenged with the clinical dose (10^4^ EID_50_) of H5N1 HPAIV (Figure 3A) were fully protected from clinical signs. Moreover, the mock inoculated group (positive control-H5N1 HPAIV) showed severe clinical signs starting from the 3rd day post-virus inoculation which were further exacerbated when chicks exposed to sub-lethal dose of H5N1 HPAIV compared to chicks in mock transgenic non-challenged (negative control) which remained healthy. Correspondingly, transgenic chicks expressing chIFITM1 were completely protected (100%) from clinical challenge without any apparent clinical disease (Figure 3A). While, the transgenic challenged group with sub-lethal dose of HPAIV showed mild disease signs with 60% protection (survival) with delayed clinical signs apparent by at least 4 days suggesting that the sub-lethal dose of H5N1 HPAI can override the overexpression of chIFITM1. Our results revealed that transgenic chicks overexpressing chIFITM1 were protected from the clinical challenge and substantially from the sub-lethal challenge, which also manifested by delayed clinical signs by at least 7 days. While as expected, 100% HPAIV mock transgenic challenged chicks (mock inoculated sub-lethal challenge) showed severe clinical signs and were culled or suddenly died due to infection within five days of challenge. Taken together, our results showed that the overexpression of chIFITM1 has a substantial impact on the appearance of the HPAIV infection clinical outcome. Likewise, chIFITM1 can protect chicks from clinical doses of influenza viruses; however, it is insufficient to completely protect chickens against the sub-lethal dose of HPAIV.

To confirm that chIFITM1 was successfully expressed in transgenic chickens, a chIFITM1-specific quantitative PCR was developed. Owing to expression of codon optimized chIFITM1 through RCASBP(A) (thus different codon usage), the PCR distinguished the transgene from endogenously expressed chIFITM1. Using this system, we found a significantly increased level of chIFITM1 in tracheal RNA obtained from transgenic chickens RCASBP(A)-chIFITM1 compared to control groups of either transgenic group with RCASBP(A)-WT or non-transgenic chickens (mock treated neg. ctrl) (*p* ˂ 0.0001) (Figure 3B) indicating the successful expression of chIFITM1.

In addition, we explored whether the increased protection in transgenic chicken with RCASBP(A)-chIFITM1 was mediated by innate immunity because of the correlation between chIFITM1-mediated induction of innate immunity [7,11]. The expression levels of four innate immune genes were evaluated and were chosen based on their antiviral expression dynamics. Our results revealed that there were no significant differences in innate immune gene expression levels between transgenic chickens (RCASBP(A)-chIFITM1) and non-transgenic (mock treated neg. ctrl) (Supplementary Table S1). These findings suggest that chIFITM1-mediated protection is not linked to enhanced secondary innate immune responses, and is specific to chIFITM1’s direct antiviral actions.

### 2.4. Virus Shedding Evaluation in Transgenic Chickens Expressing IFITM1 Challenged with HPAIV H5N1

**Figure 4:**
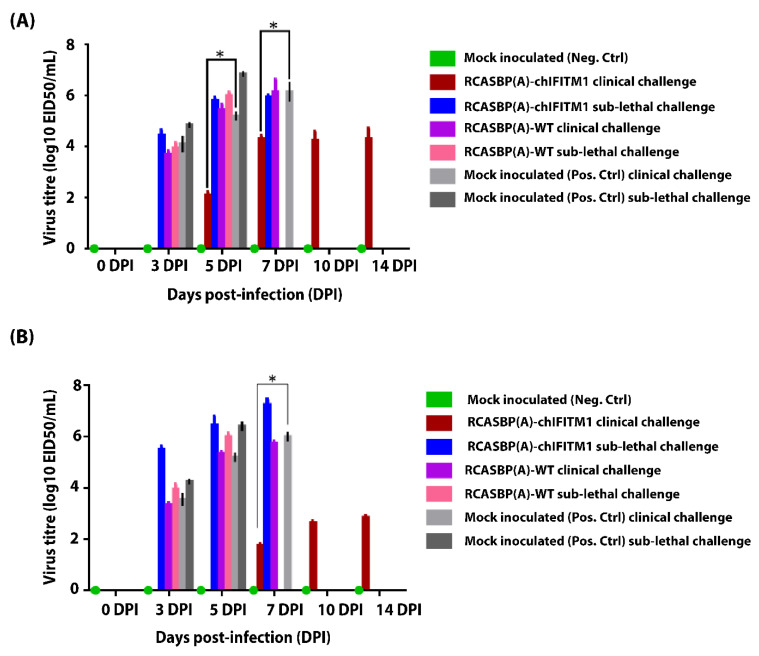
Evaluation of viral shedding from (**A**) oropharyngeal and (**B**) cloacal swabs of RCASBP(A)-chIFITM1 and RCASBP(A)-WT challenged chicks with clinical and sub-lethal doses of H5N1 HPAIV compared to mock inoculated chicks (negative and positive control groups). Each data point represents the virus titers detected in oropharyngeal and cloacal swabs on day 0, 3, 5, 7, 10, and 14 DPI. Bars represent the standard deviation means. * indicates the level of significance at *p* value < 0.05.

Cloacal and oropharyngeal swabs were collected from all groups (RCASBP(A)-chIFITM1, RCASBP(A)-WT, and mock treated (Neg. Ctrl)) before challenge and every alternative day post-clinical and sub-lethal challenges to evaluate if chIFITM1 can mediate reduction in virus shedding through oropharyngeal and cloacal routes. Our results revealed that transgenic chickens expressing chIFITM1 following clinical and sub-lethal challenge with HPAIV showed significant reduction in virus shedding in both oropharyngeal (Figure 4A) and cloacal swabs (Figure 4A) and the duration of shedding period compared to mock transgenic (Pos. Ctrl) (Figure 4A,B). These results indicate that chIFITM1 is a key factor in virus replication that contributes to lowering influenza viral shedding.

### 2.5. Virus-Induced Histopathologic Lesions Amelioration for Transgenic Chickens Expressing IFITM1

**Figure 5:**
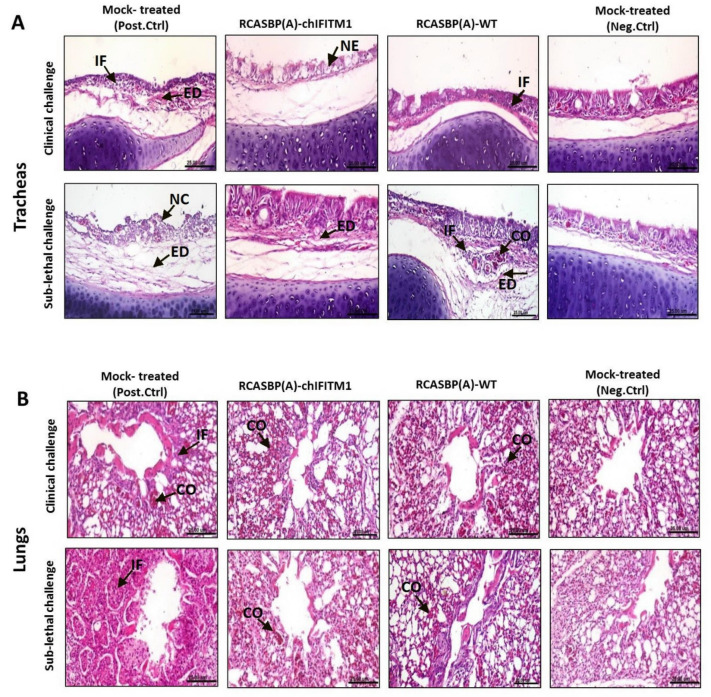
Photomicrographs representing H & E-stained sections of tracheas (**A**) and lungs (**B**) collected from RCASBP(A)-chIFITM1 and RCASBP(A)-WT challenged chicks with clinical and sub-lethal doses of H5N1 HPAIV compared to mock inoculated chicks (negative and positive control groups); showing edema (ED), focal necrosis (NE), inflammatory cells infiltration (IF), congestion (CO) (scale bar 25 μm).

Trachea and lung organs were collected from inoculated challenged chicks with clinical and sub-lethal doses followed by histopathological examination compared with non-inoculated mock controls (positive and negative control groups) to assess the level of protection offered by a stably expressing chIFITM1 in face of challenge with H5N1 HAPIV along with the induced histopathological changes. Severe histopathological alterations were noticed in tracheal sections from mock transgenic challenged control chicks (after sub-lethal challenge) including necrosis of lamina epithelialis associated with mononuclear cells infiltration and edema in the lamina propria/submucosa layer (Figure 5A). While, necrosis of some mucous secreting glands and edema in lamina propria in sections from mock transgenic challenged control chicks were observed (after clinical challenge). On the other hand, tracheas collected from transgenic chicks expressing chIFITM1 and challenged with HPAIV showed no histopathological changes (clinical challenge) while mild histopathological alterations as slight edema in lamina propria and few inflammatory cells infiltrating lamina propria (sub-lethal challenge) were observed (Figure 5A and Supplementary Table S2). Meanwhile, lungs of transgenic chicks challenged with either clinical or sub-lethal challenge showed normal parabronchus with slight congestion of pulmonary blood vessels (Figure 5B and Supplementary Table S2) while lungs of mock transgenic chicks challenged with either clinical or sub-lethal challenge showed pneumonia described by inflammatory exudate occluding the air capillaries. These findings indicate that the defense offered by the substantial expression of chIFITM1 may contribute to its antiviral activity against influenza virus replication [7,11], which collectively reflect upon the ameliorated clinical outcome and health improvement.

## 3. Discussion

With the increasing global human population, poultry production is critical for the economy and food security. Although over the years, poultry production has improved significantly by selective breeding and better genetics, threats raised by evolving and emerging pathogens have significantly increased, particularly after intensive poultry breeding systems were implemented [13]. Innate immune responses are mainly regulated by cytokines, chemokines, and interferon, which are either induced by direct viral infection or induced by intrinsic activation against pathogens. Mapping cross-species host restriction factors that determine the zoonotic potential and pathobiology of influenza viruses is fundamental to understand the molecular factors that regulate the virus-mediated innate immune responses and mechanistic observations, varying between avian and mammals. Meanwhile, additional investigation and better understanding of the alternative approaches will provide a framework against avian viral diseases and emergency of zoonotic infections such as influenza viruses by chicken immune regulation and antiviral protection [21,22].

The role of interferon stimulating genes (ISGs) against viruses of medical, zoonotic, and veterinary significance has recently been extensively explored [2]. Many of the IFITM family members have been identified in chicken, including IFITM1, IFITM2, IFITM3, and IFITM10 [11] and are differentially expressed upon stimulation by type I and type II IFNs [2]. The IFITM proteins obstruct the cytoplasmic entry for viruses. The mechanistic actions of IFITM proteins are dependent on inhibition of the virus membrane fusion because of the decreased membrane fluidity, and hence the curvature in the cell membrane outer leaflets [3]; or disruption of the homeostatic cholesterol intracellular activity by precluding the interaction of oxysterol-binding protein with the vesicle-membrane-protein associated protein A [23]. Chicken IFITM1 and IFITM3 were recently described functionally [7] although most of these studies were carried out either in cells or in ovo, which highlights the ability of chIFITM1 as an important host antiviral limitation factor

The RCAS retrovirus gene transfer method offers a simple, cheaper, and less laboratory intensive method for retroviral-mediated transgenic expression [16,24]. While a non-significant reduced body weight in transgenic chicks at hatching was observed, hatched chicks regained the weight swiftly and obtained comparable sizes to non-transgenic chicks. In the current study, we generated mosaic transgenic chickens, which stably and constitutively express chIFITM1 to further explore the in vivo antiviral function of IFITM1 against highly pathogenic avian influenza virus subtype H5N1. The transgenic chickens overexpressing chIFITM1 provided strong evidence for its ability to fully protect chickens against doses of H5N1 avian influenza viruses that cause clinical disease signs in chickens. Because of differing pressures in field environments and poultry susceptibility to environmental stresses leading to pathological symptoms caused by the influenza virus, we further investigate the impact of chIFITM1 on the predetermined clinical and sub-lethal dosages [13]. Our results revealed that chIFITM1 alone is inadequate for complete morbidity and mortality coverage when the “sub-lethal dosage” (10^5^ EID_50_) was applied. However, the clinical outcome was considerably enhanced when “clinical dose 10^4^ EID_50_” was used in transgenic chickens. Nevertheless, these finding specifically ruled out the likelihood that “clinical dose” pre-exposure could induce adaptive immune response to mask the impact of a “sub-lethal dose”. These observations clearly indicate the ability of innate immunity to protect against HPAIV. It is important to mention that the defensive function of chIFITM1 has been tested against extremely virulent viruses; highly pathogenic influenza A viruses (IAVs) that can trigger deaths rates of up to 100% in infected poultry flocks. Consequently, it is likely to be believed that chIFITM1 could have significant impacts on comparatively less virulent viruses, which cause only clinical diseases and low deaths such as H9N2 strains of influenza viruses.

Overexpression of chIFITM1 has not only alleviated the manifestation of clinical disease signs in HPAIV-infected chickens but also reduced the virus-induced pathological lesions and virus shedding. Since the RCAS-based retroviral gene transfer system is predominantly effective in organs that are rich in endothelial cells [16,22,25], we realized the complete blockage of virus shedding in trachea. This substantially reduced virus shedding correlated with the improved tracheal tissue health, which may highlight the expression and functional importance of chIFITM1 in mucosal surfaces. Meanwhile, chIFITM1 not only alleviates the clinical outcome of HPAIV infected chickens with symptoms of pathological illness, but it also decreases the pathology and viral shedding induced by viruses. As the RCAS propagation mechanism focus is primarily successful in endothelial cell-rich tissues, that might explain why the lower virus replication and shedding in transgenic chicken overexpressed chIFITM1 compared to wild type chickens. This decreases virus accumulation in tandem with the greater protection of tracheal tissues and may demonstrate the presence and functional value of chIFITM1 on mucosal surfaces. To conclude, the antiviral activities of chIFITM1 against HPAI H5N1 was defined by the use of the animal transgenic model. These findings indicate the ability of the innate immune system to impart tolerance to viruses in chicken and provide proof of the capacity to produce virus-resistant transgenic chickens for food protection and to inhibit the spread of zoonotic viruses to humans. In addition, gaining more understanding of the genetic factors that determine the susceptibility of poultry to avian influenza viruses will help to diminish risks to animal and human health via outbreak preparedness, enhancing food security, and animal health and welfare. However, understanding these factors will not only help to understand how influenza viruses evolve but also provide evidence as to how such a host spectrum contributes to circulation of influenza viruses in chickens and their potential risk to humans.

## 4. Materials and Methods

### 4.1. Ethics Statement

All animal studies and procedures were carried out in strict accordance with the guidelines of the Animal Ethics Committees, Department of Poultry Viral Vaccines, Veterinary Serum and Vaccine Research Institute (VSVRI), Agriculture Research Centre (ARC), Egypt. The study was conducted according to the guidelines of the Declaration of the Veterinary Serum and Vaccine Research Institute (VSVRI) and approved by the Institutional Review Board (VSVRI-20180206).

### 4.2. Cells, Viruses and Antibodies

DF1 cells (chicken fibroblast line; ATCC CRL-12203) were cultured in Dulbecco’s modified Eagle’s medium (DMEM) (Gibco, Carlsbad, CA 92010, USA) supplemented with 10% inactivated fetal bovine serum (FBS) 120 (Gibco), 2 mM l-glutamine (Gibco), and 100 U/mL penicillin/streptomycin (Gibco) at 37 °C in 5% CO_2_. Influenza A virus strain A/chicken/Egypt_128s_2012 (clade 2.2.1.2) (accession number: JQ858485.1) was propagated in 9-day-old specific pathogen free (SPF) chicken eggs and the median egg infectious doses 50 (EID_50_) were determined in SPF eggs using the Reed and Muench method [26]. AMV-3C2-S (gag) antibodies were purchased from Hybridoma Bank of Iowa, University of Iowa. The α-flag antibodies for the detection of FLAG tag-fused chIFITM1 were purchased from Sigma (Sigma-Aldrich, MA, USA). Alexa-fluor 568 and 488 secondary antibodies were purchased from Invitrogen (Carlsbad, CA, USA).

### 4.3. Construction and Rescue of RCAS Viruses Expressing chIFITM1

The open reading frame of chIFITM1 codon-optimized and chemically synthesized in-fusion with Flag-tag and sub-cloned to an improved form of RCASBP(A)-∆F1 (kindly provided by Stephen H. Hughes, National Cancer Institute, Maryland USA) using *ClaI* and *MluI* restriction sites. This restriction digestion excised the *src* gene and replaced it with chIFITM1 while maintaining the splice accepter signals. This new vector was designated as RCASBP(A)-chIFITM1. In order to generate reporter RCASBP(A) system, the GFP coding sequence was cloned between *ClaI* and *MluI* and the resulting plasmid was labelled as RCASBP(A)-eGFP [23]. The sequence integrity and orientation were confirmed by Sanger’s sequencing. To rescue recombinant RCASBP(A) retroviruses, we followed previously described methods [23]. Briefly, DF1 cells transfected with each of the RCASBP(A)-eGFP and RCASBP(A)-chIFITM1 plasmids using Lipofectamine 2000 in Opti MEM with a predetermined optimized ratio of 1:3 (Invitrogen, Carlsbad, CA, USA). Media were changed 6 h post transfection and replaced DMEM supplemented with 5% FCS and 1% penicillin/streptomycin for 48 h. Cells were expanded until the desired number of cells (10^6^ cells/egg) was achieved.

### 4.4. Confocal Microscopy

Expression of the reporter gene (GFP) was monitored using fluorescence microscopy (Figure S1) whereas replication efficiencies of chIFITM1 expressing retroviruses were assessed by staining the gag protein of RCASBP(A) and chIFITM1-Flag tag. DF1 cells grown on coverslips in 24-well plates, were infected with retroviruses (RCASBP(A)-chIFITM1) for 48 h. Cells were then fixed for 1 h using 4% paraformaldehyde and permeabilized using 0.01% Triton-X100 before incubation with primary antibodies raised against either Flag tag, gag protein of retroviruses, or both. Afterwards, cells were incubated with corresponding secondary antibodies for 2 h at room temperature. Cell nuclei were stained with 4′, 6-diamidino-2-phenylindole (DAPI), and the images were taken using a Zeiss confocal laser-scanning microscope (Zeiss, Kohen, Germany). The confocal images were taken with 40× and 63× high numerical-aperture oil immersion objective lenses on an upright Zeiss LSM800 confocal microscope. The image size was set 1024 × 1024 pixels. To eliminate inter-channel cross talk, multitrack sequential acquisition settings were used. A 568 nm diode-pumped solid-state laser and an argon ion laser’s 488 nm line were used for excitation. Zeiss Zen control software, which provides numerous viewing features for the observation and creation of high-quality confocal images, was used to establish optimized emission detection bandwidths.

### 4.5. Generation of Transgenic Chickens and H5N1 HPAIV Challenge

SPF eggs were acquired from a local supplier in co-operation with the Department of Poultry Viral Vaccines, Veterinary Serum and Vaccine Research Institute (VSVRI), Agriculture Research Centre (ARC), Egypt. Mosaic-transgenic chicken embryos were generated by inoculation of 10^6^ RCASBP(A)-chIFITM1 or empty RCASBP(A)-WT infected DF-1 cells into SPF chicken eggs through the intra-yolk sac using 24G needles at day 2 post-embryonation (ED2). Eggs were fixed for 2 h post-inoculation before incubation at 37 °C with 60–80% humidity in a rotating incubator (twice daily). Transgenic embryos were allowed to hatch naturally at 21 days of incubation (ED21) (Figure 2A). Each group of transgenic chickens was housed separately in containment level 3 isolators. Food and water were provided ad libitum, and general animal care was provided by the animal house staff as required.

The virus dosage optimization (clinical and sub-lethal doses) for HPAIV H5N1 was carried out in our previously study [13]. A total of 20 RCASBP(A)-chIFITM1, 20 RCASBP(A)-WT transgenic chicks, and 15 mock-inoculated chicks (positive control) were challenged with 10^4^ EID_50_ H5N1 HPAIV (clinical dose) 12 days post-hatching (PH12). On the other hand, 10 chicks were kept as a naïve negative control group (non-inoculated-non challenged, inoculated with PBS). Before second challenge with the sub-lethal dose (10^5^ EID_50_ H5N1 HPAIV) on day 20 post-hatching (PH20), three chicks from all groups were sacrificed for histopathological examination. All birds in all groups were monitored for the following 15 days to monitor the appearance of clinical signs, weight gain (Figure 2C), feed intake (Figure 2D) and water intake (Figure 2E), and mortalities in all groups. The experiment was terminated on day 35 (PH35) and all remaining chicks were euthanized.

### 4.6. Confirmation of chIFITM1 Expression and Quantitative Assessment of the Chicken Antiviral Immune Responses

Total RNA was extracted from tracheas and lungs, which were collected from transgenic (RCASBP(A)-chIFITM1) and non-transgenic chickens (mock treated neg. ctrl) using TRIzol reagent (Invitrogen, Carlsbad, CA, USA). A total of 150 ng of RNA was used in the PCR reactions using SuperScript III Platinum SYBR Green One-Step qRT-PCR Kit (Invitrogen, Carlsbad, CA, USA) as described earlier [24]. The abundance of specific chIFITM1 mRNA was compared to the 28S rRNA. The reactions were run using a CFX96 Real-Time PCR machine (Bio-Rad, Hercules, CA, USA) and the data were analyzed using the ddCt method [27].

In order to determine the expression of innate immune genes, total RNA was extracted as described above using TRIzol reagents (Invitrogen, Carlsbad, CA, USA). Invitrogen SuperScript III Platinum One-Step qRT-PCR Kit (Invitrogen, Carlsbad, CA, USA) was used for quantification of the abundances of specific innate immune genes’ mRNA in tracheas of transgenic chickens with RCASBP(A)-chIFITM1, non-transgenic (mock treated pos. ctrl) chicks challenged with HPAIV H5N1, and negative control birds compared to corresponding 28S rRNA (housekeeping gene) and the average fold changes were determined as provided in Supplementary Table S1. Primers for innate immune genes are provided in Supplementary Table S1.

### 4.7. Virus Shedding and Histopathology

Cloacal and oropharyngeal swabs were collected separately, placed in virus transport medium, filtered through a 0.2 µm filter and then aliquoted and stored at −70 °C until all samples were collected before analysis using hemagglutination assay as previously described [28]. Selections of tissues including trachea and lung were collected and fixed at room temperature for 48 h by immersion in 10% neutral buffered formalin followed by paraffin wax embedding. The 5 µm tissue sections were stained using hematoxylin and eosin stain before examination under light microscope for any microscopic lesions. Quantitative scoring for histopathological lesions for the trachea and lungs were evaluated on a scale from 0 to 3 based on the lesion severity grade (mild, moderate, and severe) as follow: 0 = no changes, 1 = mild, 2 = moderate, and 3 = severe [29].

### 4.8. Statistical Analysis

Pairwise comparisons of challenged (clinical and sub-lethal doses) and control groups (positive and negative) were performed using Student’s *t*-test. Kaplan–Meier analysis was performed to calculate the survival rates. Two-tailed Student’s *t*-test and one-way analysis of variance (ANOVA) were used to determine differences between groups. Statistical significance is shown with values of *p* < 0.05. All data were represented as the mean ± standard deviation (SD). Statistical analyses were conducted using GraphPad Prism 7 (GraphPad Software, La Jolla, CA, USA).

## Data Availability

Not applicable.

## Supplementary Materials

The following are available online at https://www.mdpi.com/article/10.3390/ijms22168456/s1, Figure S1. Generation and rescue of recombinant retroviruses expressing marker gene (eGFP). Supplementary data 1 file represents the statistical analysis for hatchability between different inoculated groups. Supplementary data file 2 represents the statistical analysis for the average body weight between different inoculated groups during the experiment. Supplementary data file 3 represents the statistical analysis for the average feed intake between different inoculated groups during the experiment. Supplementary data file 4 represents the statistical analysis for the average water consumption between different inoculated groups during the experiment. Supplementary Table S1: Expression of innate immune genes in transgenic and non-transgenic chicken challenged with HPAI H5N1. Supplementary Table S2: Histopathological lesion scores for tracheas and lungs of different experimental groups.
