# Supplementary material for: Transgenic Chicks Expressing Interferon-Inducible Transmembrane Protein 1 (IFITM1) Restrict Highly Pathogenic H5N1 Influenza Viruses
Source: Int J Mol Sci. 2021 Aug 6;22(16):8456. doi: 10.3390/ijms22168456 (PMC8395118; doi:10.3390/ijms22168456)
Supplement: Supplementary file 1 [file ijms-22-08456-s001.zip › Table S1_innate immune genes.pdf]

Supplementary Table S1. Expression of innate immune genes in transgenic and non-transgenic chicken challenged with HPAI H5N1.

Innate immune genes	Sense & Anti-sense Primers	Fold change		
		Non-transgenic (Mock treated Neg. Crtl)	Transgenic (RCASBP(A)-chIFITM1	Significance (*P value*)**
Mx	5′-CACTGCAACAAGCAAAGAAGGA-3′<br>5′-TGATCAACCCCACAAGGAAAA-3′	86.0	89.0	NS= 0.83
IFN-β	5′-CCTCCAACACCTCTTCAACATG-3′<br>5′-TGGCGTGCGGTCAAT-3′	35.0	39.0	NS= 0.67
Viperin	5′-TGCTTAAGGAGGCGGGAATG-3′<br>5′-CAGCTGGCCTACAAATTCGC-3′	19.0	18.0	NS= 0.75
IFI35	5′-TGGTCCGCTATCCTCTGTCA-3′<br>5′-CTCGAGTGAGCCCAATCTCC-3′	33.0	37.0	NS= 0.83
28S	5′-GGCGAAGCCAGAGGAAACT-3′<br>5′-GACGACCGATTTGCACGTC-3′	NA	NA	NA

*Fold change represents average of triplicate repeats run on samples, compared to corresponding 28S and is rounded to full number. ** *P values* >0.05 were considered non-statistically significant (NS).
