# Supplementary material for: Transgenic Chicks Expressing Interferon-Inducible Transmembrane Protein 1 (IFITM1) Restrict Highly Pathogenic H5N1 Influenza Viruses
Source: Int J Mol Sci. 2021 Aug 6;22(16):8456. doi: 10.3390/ijms22168456 (PMC8395118; doi:10.3390/ijms22168456)
Supplement: Supplementary file 1 [file ijms-22-08456-s001.zip › Table S2_PM lesions score.pdf]

Supplementary Table S2. Histopathological lesion scores for tracheas and lungs of different experimental groups.

Histopathological lesions			Mock-treated (Neg. Ctrl))	Clinical Challenge			Sub-lethal Challenge		
				Mock-treated (Post. Ctrl	RCASBP(A)-chIFITM1	RCASBP(A)-WT	Mock-treated (Post. Ctrl)	RCASBP(A)-chIFITM1	RCASBP(A)-WT
**Trachea**	Necrosis of lamina epithelialis		0.0±0.0	1.4±0.5	0.2±0.4	1.1±0.5	2.6±0.5	0.4±0.5	2.4±0.5
	Necrosis of some mucous secreting glands		0.0±0.0	1.6±0.5	0.4±0.5	1.4±0.5	2.6±0.5	0.4±0.5	1.8±0.4
	Edema in lamina propria/sub mucosa		0.0±0.0	2.6±0.5	0.2±0.4	2.2±0.4	2.6±0.5	0.4±0.5	2.4±0.5
	Inflammatory cells infiltrating		0.0±0.0	2.2±0.4	0.0±0.0	2.0±0.7	2.6±0.5	0.0±0.0	2.2±0.4
**Lungs**	Congestion of pulmonary blood vessels		0.0±0.0	2.6±0.5	0.6±0.5	2.2±0.4	3.0±0.0	0.8±0.4	2.6±0.5
	Inflammatory exudate		0.0±0.0 [a]	2.0±0.0	0.0±0.0	1.2±0.8	2.40±0.5	0.0±0.0	1.8±0.8

Data shown as mean ± SD; one-way analysis of variance (ANOVA) were used to determine differences between groups. Statistical significance is shown with values of $p < 0.05$
