# Supplementary figures and images for: Transgenic Chicks Expressing Interferon-Inducible Transmembrane Protein 1 (IFITM1) Restrict Highly Pathogenic H5N1 Influenza Viruses
Source: Int J Mol Sci. 2021 Aug 6;22(16):8456. doi: 10.3390/ijms22168456 (PMC8395118; doi:10.3390/ijms22168456)

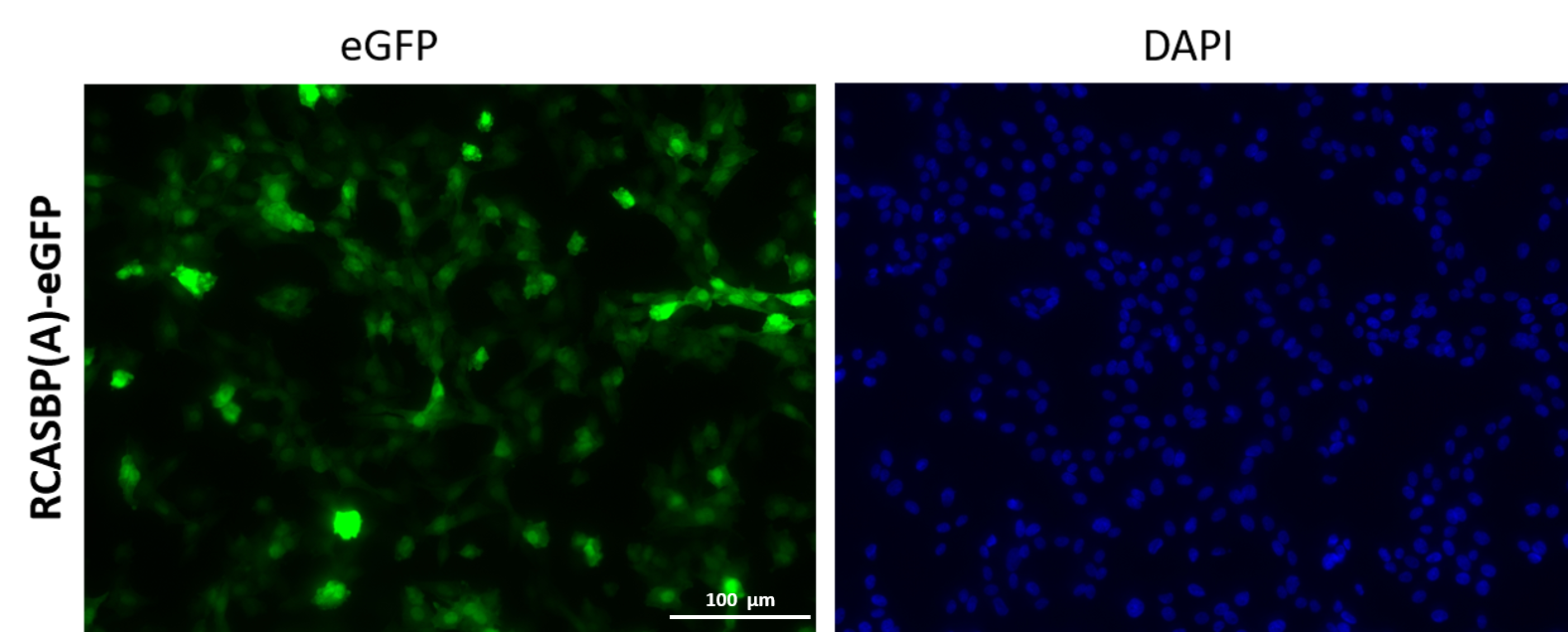

Supplement: Supplementary file 1 [file ijms-22-08456-s001.zip › Supplementary Figure S1.tif]
